# *In Vivo* MRI Tracking of Mesenchymal Stromal Cells Labeled with Ultrasmall Paramagnetic Iron Oxide Particles after Intramyocardial Transplantation in Patients with Chronic Ischemic Heart Disease

**DOI:** 10.1155/2019/2754927

**Published:** 2019-11-14

**Authors:** Anders Bruun Mathiasen, Abbas Ali Qayyum, Erik Jørgensen, Steffen Helqvist, Annette Ekblond, Michael Ng, Kishore Bhakoo, Jens Kastrup

**Affiliations:** ^1^Cardiac Stem Cell Center and Catheterization Laboratory, The Heart Centre, Rigshospitalet, University of Copenhagen, Copenhagen, Denmark; ^2^Translational Imaging Group, Singapore Bioimaging Consortium, Agency for Science, Technology and Research, Singapore, Singapore

## Abstract

**Background:**

While regenerative stem cell therapy for ischemic heart disease has moved into phase 3 studies, little is still known about retention and migration of cell posttransplantation. In human studies, the ability to track transplanted cells has been limited to labeling with radioisotopes and tracking using nuclear imaging. This method is limited by low resolution and short half-lives of available radioisotopes. Longitudinal tracking using magnetic resonance imaging (MRI) of myocardial injected cells labeled with iron oxide nanoparticles has shown promising results in numerous preclinical studies but has yet to be evaluated in human studies. We aimed to evaluate MRI tracking of mesenchymal stromal cells (MSCs) labeled with ultrasmall paramagnetic iron oxide (USPIO) nanoparticles after intramyocardial transplantation in patients with ischemic heart disease (IHD).

**Methods:**

Five no-option patients with chronic symptomatic IHD underwent NOGA-guided intramyocardial transplantation of USPIO-labeled MSCs. Serial MRI scans were performed to track labeled cells both visually and using semiautomated T2∗ relaxation time analysis. For safety, we followed symptoms, quality of life, and myocardial function for 6 months.

**Results:**

USPIO-labeled MSCs were tracked for up to 14 days after transplantation at injection sites both visually and using semiautomated regional T2∗ relaxation time analysis. Labeling of MSCs did not impair long-term safety of treatment.

**Conclusion:**

This was a first-in-man clinical experience aimed at evaluating the utility of MRI tracking of USPIO-labeled bone marrow-derived autologous MSCs after intramyocardial injection in patients with chronic IHD. The treatment was safe, and cells were detectable at injection sites up to 14 days after transplantation. Further studies are needed to clarify if MSCs migrate out of the injection area into other areas of the myocardium or if injected cells are washed out into the peripheral circulation. The trial is registered with ClinicalTrials.gov NCT03651791.

## 1. Introduction

Regenerative treatment with stem cells in chronic ischemic heart disease (IHD) is a relatively new treatment modality.

Numerous clinical trials using different cell types and delivery methods for both acute and chronic ischemic heart disease have been conducted. Results have been diverging. In Cochrane surveys, no beneficial effects of cell therapy in general were found for acute ischemic heart disease (myocardial infarction) [1]. In chronic ischemic heart disease and heart failure, there is some evidence that cell therapy might reduce mortality and clinical symptoms and improve heart pump function [2]. The vast majority of trials in these surveys used intracoronary infused mononuclear cells.

Focus has since changed to more homogenous cell types, such as mesenchymal stromal cells (MSCs) delivered by direct intramyocardial injection. In several randomized clinical trials, MSCs have been injected directly into viable myocardium in the border zone of scar tissue in patients with chronic ischemic heart disease and heart failure [3–8]. Results from these trials have been generally positive with improvements in heart pump function, reduction of scar tissue, reduced clinical symptoms, and improved quality of life. Several, larger multicenter trials are ongoing to confirm the encouraging results.

Little is still known about retention and migration of cell posttransplantation. Such studies are imperative in assessing the efficacy and safety of stem cell-based therapy.

In human studies, the ability to track transplanted cells has been limited to labeling with radioisotopes and subsequent tracking using nuclear imaging. These methods are limited by low spatial resolution and short half-lives of clinically approved radioisotopes, ranging from minutes to hours, and therefore only allowing short-term tracking of the cells. Other limitations are exposure to ionizing radiation and nontarget signal leakage [9, 10].

Tracking cells labeled with iron oxide nanoparticles using magnetic resonance imaging (MRI) offer higher spatial and temporal resolution in combination with higher soft tissue contrast, without exposing patients to ionizing radiation. This method has been utilized *in vivo* in different animal models of myocardial infarction (MI). In the majority of these studies, the labeled cells were tracked in the myocardium for up to 16 weeks after transplantation [11–20]. In contrast, a few studies showed that the cells were only retained in the heart for few hours [21, 22].

*In vivo* tracking of iron oxide-labeled cells has not yet been utilized in a clinical cardiovascular setting, but it has been used successfully in several noncardiovascular clinical studies [23–29]. Moreover, labeling of cells with iron oxide nanoparticles has been demonstrated to be clinically safe and does not affect cellular function of the labeled cells [30].

MRI T2∗-weighted imaging is a method which is influenced by inhomogeneities in the magnetic field, such as high iron overload thalassemic cardiomyopathy. The method is also useful to depict iron particles introduced to the heart.

Iron oxide particles of varying size have been used for cell tracking. The smallest of these are ultrasmall superparamagnetic iron oxide (USPIO) particles (5-40 nm). In practice, USPIO particles have been shown to be more suitable than larger iron oxide particles for tracking of nonphagocytic cells, such as MSCs, due to higher cellular uptake [31–33]. Moreover, to successfully internalize iron particles into nonphagocytic cells such as MSCs, it is necessary to conjugate the iron particles with a membrane translocation agent such as TAT-peptide [34].

In preparation for the present clinical study, we carried out several preclinical studies. In one study, we compared MSCs labeled with the USPIO particles used in the present study with unlabeled MSCs. We assessed the ultrastructure of the cells using electron microscopy and found no differences to cell ultrastructure after USPIO labeling. In addition, we compared cell viability, phenotype, and proliferation capacity and found no differences after USPIO labeling [35]. In another study, we compared the MRI signal of USPIO-labeled cells using different labeling doses and labeling (incubation) times and found that the optimal labeling dose was 10 *μ*g USPIO particles per 10^5^ MSCs. In the same study, we assessed MRI detection limits in porcine hearts in which different quantities of USPIO-labeled MSCs were injected. As few as 250.000 USPIO-labeled cells were detectable in the following image analysis [36].

This was a first-in-man clinical experience aimed at evaluating the utility of MRI tracking of USPIO-labeled bone marrow-derived autologous MSCs after intramyocardial injection in patients with chronic IHD.

## 2. Materials and Methods

### 2.1. Study Overview

The study was a single-center, nonrandomized, pilot study performed at Rigshospitalet, University of Copenhagen, Denmark. The study protocol complied with the Declaration of Helsinki and was approved by the Danish National Ethical Committee (j.no: 1090356) and Danish Medicines Agency (j.no: 2013022184 and EudraCT-no: 2012-004047-71). The study was registered at ClinicalTrials.gov (Identifier: NCT03651791).

Manufacturing of GMP- (Good Manufacturing Practice-) grade USPIOs was done at Singapore Bioimaging Consortium, Agency for Science, Technology and Research (A∗STAR), Singapore. Protocols for manufacture and labeling of cells with USPIO were developed in-house and were used according to authorization for human medicinal products issued by the Danish Health and Medicines Authority. The study data were monitored continuously during the study period by the regional Good Clinical Practice unit.

### 2.2. Patient Population

Key inclusion criteria were patients aged 30-80 years with chronic stable IHD with at least one significant stenosis of a larger coronary artery with no option for either percutaneous coronary intervention (PCI) or coronary artery bypass grafting (CABG). Patients were on maximal tolerable medication and had moderate to severe symptoms and classified New York Heart Association (NYHA) Classes II-IV or Canadian Cardiovascular Society (CCS) Classes II-IV. Major exclusion criteria were acute coronary syndrome, stroke, or transitional cerebral ischemia within 6 weeks, revascularization within 4 months, moderate or severe valvular disease, severe chronic pulmonary disease, morbid obesity, and history of cancer within 5 years. All patients provided written informed consent and were followed for 6 months. For details on inclusion and exclusion criteria and follow-up program, see Supplementary Materials.

### 2.3. Objectives

The primary objective was positive identification of USPIO-labeled MSCs on day 0 after myocardial injection using MRI. Secondary objectives were the identification of the labeled cells using MRI on day 1 and thereafter at 1, 2, 4, 8, and 26 weeks after injection. For safety, we followed symptoms, quality of life, and myocardial function.

### 2.4. Bone Marrow Cell Preparation and Culturing

The isolation and culture expansion of the MSCs from bone marrow have previously been described in detail [37, 38]. Release criteria were sterility, viability, and MSC morphology. Cell expansion was limited to two culture passages. Expansion for only two passages was chosen as a guarantor of preserved primary cell features overriding total number of cells. Minimal criteria for defining MSCs according to the International Society for Cellular Therapy (ISCT) position statement were applied [39]. The culture medium was tested for bacteria, yeast, and mycoplasma 1 week before and on the day of transplantation.

### 2.5. USPIO Preparation and Labeling Procedure

USPIO nanoparticles coated with dextran and conjugated with TAT-peptide (IODEX-TAT; 15–20 nm) were prepared under GMP conditions using the method described by Josephson et al. [9, 34]. The final iron concentration was 2.5 mg/mL, and the solution was sterilized by filtration through 0.22 *μ* and gamma irradiation prior to use.

At 21 hours prior to cell transplantation, labeling of approximately 10 × 10^6^ of the cultured MSCs was initiated. As described previously [36], MSCs were labeled by incubation with IODEX-TAT nanoparticles at a concentration of 10 *μ*g iron per 10^5^ cells/mL complete medium for 21 hours at 37°C in humidified incubator at 5% CO_2_. The cells were then washed 3 times in PBS (Phosphate-Buffered Saline, Invitrogen, Austria) and harvested with TrypLe Select (Invitrogen, Austria) and centrifuged 5 min at 300 g. After centrifugation, the cells were resuspended in PBS; cell numbers and cellular viability were determined by propidium iodide staining using a NucleoCounter NC-100 (Chemometec, Denmark). IODEX labeling was confirmed by Prussian blue cytology staining for iron on a sample of the cell culture.

### 2.6. Cell Transplantation

LV mapping was performed with the NOGA-XP system and intramyocardial injections with Myostar injection catheters (Biologics Delivery Systems Group, Johnson & Johnson, USA) [40].

A total of 3-4 injections of 0.2 mL USPIO-labeled MSCs and 9-15 additional injections of 0.2 mL unlabeled MSCs were made in the border zone between viable (unipolar voltage > 6 mV) and nonviable myocardia (unipolar voltage < 6 mV).

**Figure 1:**
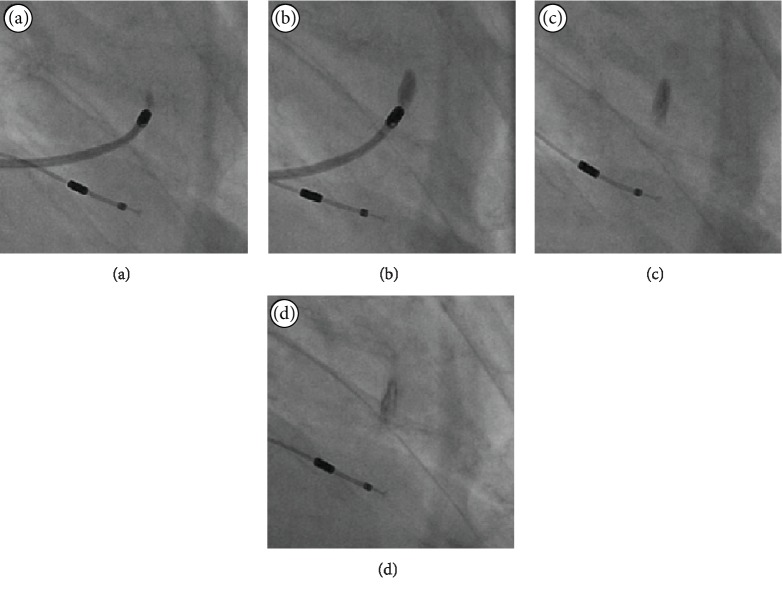
NOGA injection dispersion pattern. X-ray images demonstrate the immediate dispersion pattern of an injected solution using the NOGA system, in this instance, iodine contrast. Images (a) and (b) depict the start and end of injection over approximately one minute, while images (c) and (d) after 2 and 3 minutes.

Figure 1 demonstrates the immediate dispersion pattern of an injected solution using the NOGA injection catheter. In this instance, it is the iodine contrast injected into the myocardium and X-ray images. This procedure was not part of the current study.

### 2.7. Magnetic Resonance Imaging

MRI was performed on a clinical 1.5 T scanner (Magnetom Avanto; Siemens, Germany) using a body matrix coil. Two protocols were used. The first was a standard cardiac MRI with LV short-axis cine images of the entire left ventricle. The standard cardiac MRI was performed at baseline and after 12 and 26 weeks. The second protocol was a standard thalassemia T2∗-weighted gradient echo (GRE) black blood sequence, with a repetition time of (TR) 200 ms, a flip angle of 20°, a matrix of 116 × 128, a field of view (FOV) of 270 × 300 mm, and a slice thickness of 5 mm with no gaps between slices. For each slice, this protocol produced 12 images at the exact same time in middiastole with different echo times (TE) (1.29, 3.14, 5.04, 6.94, 8.84, 10.74, 12.64, 14.54, 16.44, 18.34, 20.24, 22.14 ms). The T2∗ protocol was performed at every MRI session.

### 2.8. Analysis of MRI Images

Cine images were analyzed with the CVI42 postprocessing tool (Circle Cardiovascular Imaging, Canada). Endocardial and epicardial borders were traced manually in the end diastole and end systole, and the mitral plane was set to define the basal border of the LV.

T2∗ images were analyzed with the VIRTUE postprocessing tool (Diagnosoft Inc., USA). For each slice, endocardial and epicardial borders of the LV were traced manually. Then, the anterior right ventricle insertion point was positioned and a 24-segment mesh was automatically created covering the myocardium of the LV. For each segment of each slice, the T2∗ relaxation time (ms) was calculated by the software. For each MRI performed after baseline, the difference between baseline values and new values was calculated. This was done to adjust for background noise. Bullseye plots of the regional differences in T2∗ values were created using MATLAB (The MathWorks, Inc., USA).

### 2.9. Statistics

Statistical analyses were done using SPSS 24 (IBM Corp., Armonk, NY, USA). The nonparametric Wilcoxon signed rank test was used for all analyses of clinical data. A two-sided *p* value of <0.05 was considered statistically significant.

## 3. Results

### 3.1. Patients and MSCs

**Figure 2:**
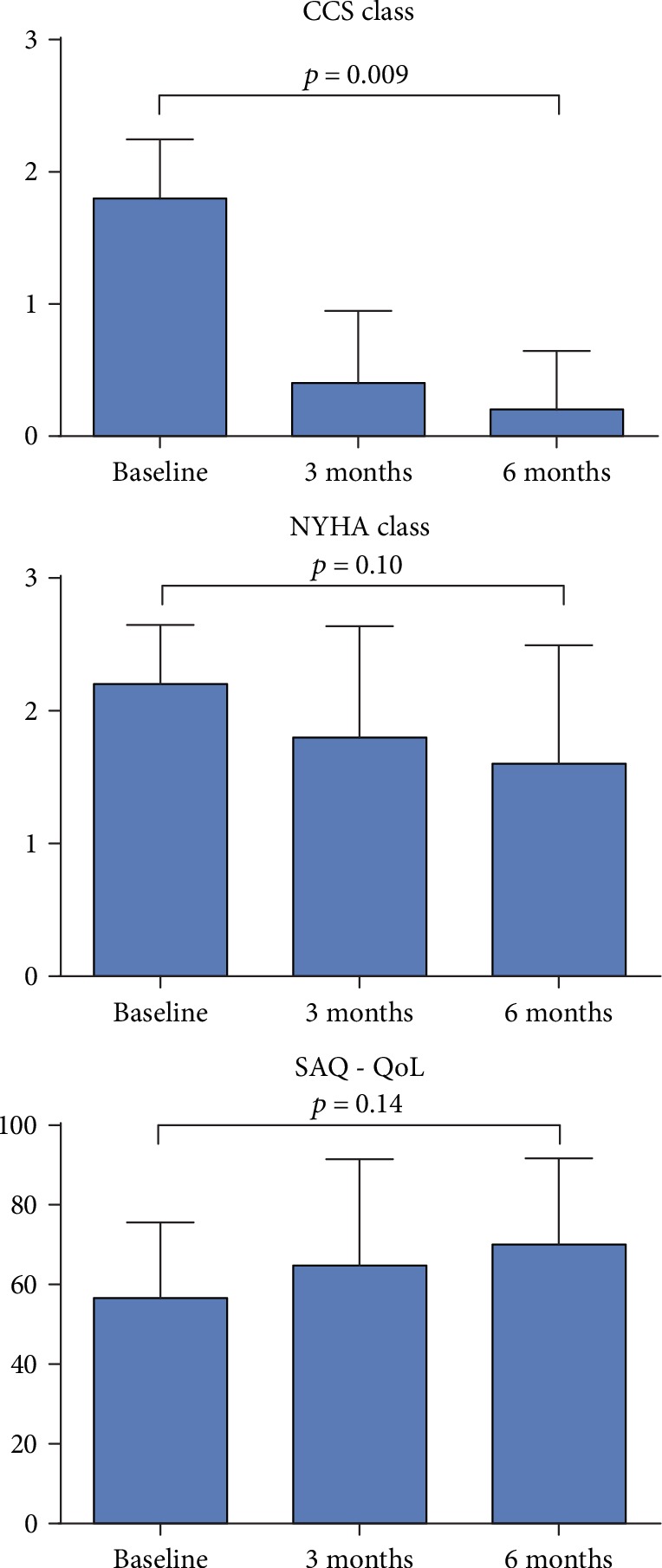
Difference in clinical symptoms and quality of life. Difference in angina symptoms (CCS class), shortness of breath symptoms (NYHA class), and quality of life score (SAQ) from baseline to 6 months after treatment. Bar values are mean values + standard deviation (SD). CCS = Canadian Cardiovascular Society; NYHA = New York Heart Association; SAQ = Seattle Angina Questionnaire.

**Figure 3:**
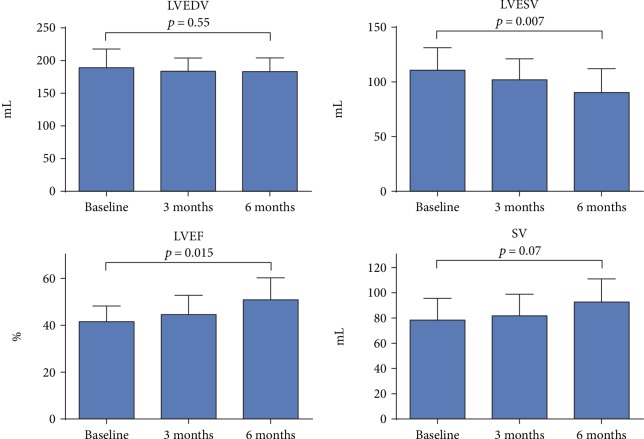
Difference in left ventricular systolic function. Differences in MRI measurements of left ventricle (LV) end-diastolic volumes (LVEDV), LV end-systolic volumes (LVESV), LV ejection fractions (LVEF), and stroke volumes (SV) at baseline and 3 and 6 months after treatment. Bar values are mean values + standard deviation (SD). MRI = magnetic resonance imaging.

Five no-option patients with chronic IHD and moderate to severe cardiac symptoms were included into the trial, and all five patients were treated successfully. Baseline characteristics and clinical outcomes are shown in Figures 2 and 3 and in detail in Supplementary Materials.

MSCs were successfully culture expanded under good manufacturing practice conditions. Patients were treated with the cells after two culturing passages. All patients were treated with 3-4 injections of USPIO-labeled MSCs and 9-15 injections of unlabeled MSCs comprising a mean of 24 ± 19 × 10^6^ (range 12 − 57 × 10^6^) USPIO-labeled MSCs and 306 ± 175 × 10^6^ (range 101 − 496 × 10^6^) unlabeled MSCs. There were no incidences of contaminations with bacteria, yeast, or mycoplasma. All cultures had normal MSC morphology, and IODEX labeling was confirmed in all cultures.

There was one serious adverse event, cardiac tamponade, related to the NOGA procedure. Pericardiocentesis was performed immediately and the clinical course thereafter was uneventful. The patient completed the follow-up. No other serious adverse events related to the treatment or in particular to the labeling of MSCs with iron oxide particles were observed.

All patients completed all follow-up visits.

### 3.2. Cardiac MRI

All patients had cardiac MRI scan performed at baseline and days 0, 1, 3, and 7 and 2 weeks, 1 month, 3 months, and 6 months after treatment. Functional images to assess heart pump function were done at baseline and after 3 and 6 months. T2∗ images were done at baseline; at days 0, 1, 3, and 7; and after 2 weeks and after 1 month. The 2-month T2∗ scan was omitted for all patients as there were no visible signs of USPIO-labeled cells in any of the patients after 1 month.

**Figure 4:**
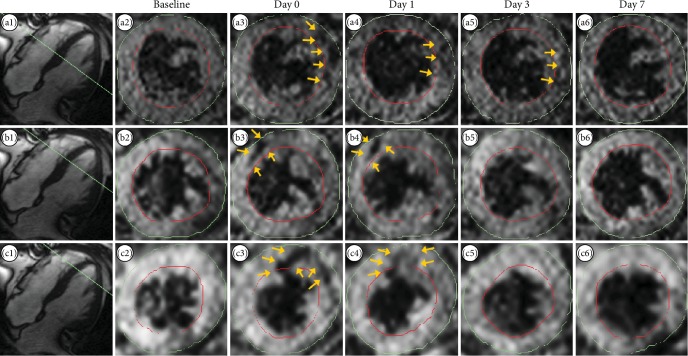
T2∗ images. T2∗ images from one representative patient. Images a1, b1, and c1 show the longitudinal position of respective short-axis images (green lines). Images a2–a6, b2–b6, and c2–c6 show serial short-axis images at the same three longitudinal positions (a, b, c) at baseline, day 0, day 1, day 3, and day 7 after injection. The yellow arrows point to hypointense areas in the images suspected to point out injected USPIO-labeled MSCs (slice thickness 5 mm). USPIO = ultrasmall paramagnetic iron oxide; MSC = mesenchymal stromal cell.

For T2∗ imaging, hypointense areas were observed in the myocardium in all patients at day 0 after injection. The hypointense areas correlated well with the areas of cell injection. Hypointense areas were visible between 1 and 14 days after injection. After this period, there were no visible signs of injected USPIO-labeled cells. In Figure 4, selected MRI scan images from a representative patient are shown. These images were acquired with TE of 22.14 ms. Images in each row show serial left ventricular short-axis images in the same longitudinal position at baseline and the first week of follow-up. In the upper row, a midventricular hypointense area was observed in the lateral part of the ventricle (arrows) for three days after injection. In the middle row, a midventricular-apical hypointense area was observed in the anteroseptal part of the ventricle up till 1 day after injection, and in the lower row, an apical hypointense area was observed in the anterior part of the ventricle up till 1 day after injection.

Short-axis cine MRI imaging demonstrated improvements from baseline to 6-month follow-up in heart pump function. This is shown in Figure 3 and in detail in Supplementary Materials.

**Figure 5:**
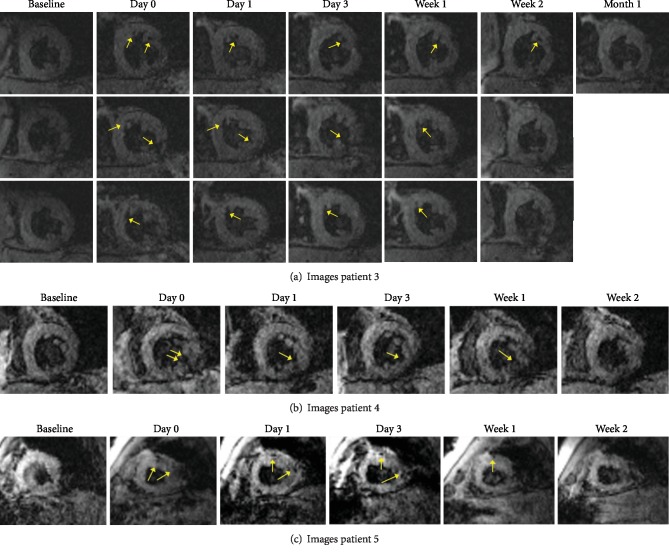
MRI images of patients 3–5. T2∗ images from patient 1 are in the main text. Images from patient 2 are not shown due to cardiac tamponade. The yellow arrows point to hypointense areas in the images suspected to point out injected USPIO-labeled MSCs.

Images from additional patients are shown in Figures 5(a)–5(c).

### 3.3. T2∗ Image Analysis and NOGA Injection Map

**Figure 6:**
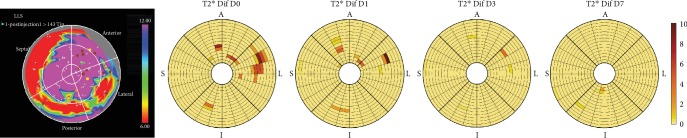
NOGA injection points and T2∗ relaxation time difference maps. Images are from the same patient as Figure 4. The left image shows the NOGA™ injection map of the patient's left ventricle after injections. The colors represent endocardial voltage in mV with the lowest voltages of nonviable tissue (red). Injection points of USPIO-labeled MSCs are shown as yellow points on the map, and brown points represent injection points of unlabeled MSCs. The images on the right shows regional differences in T2∗ relaxation (ms) time from baseline to day 0 (D0), baseline to day 1 (D1), baseline to day 3 (D3), and baseline to day 7 (D7). A = anterior; L = lateral; I = inferior; S = septum; USPIO = ultrasmall paramagnetic iron oxide; MSC = mesenchymal stromal cell.

Differences in T2∗ relaxation times between baseline and follow-up scans revealed decreases in T2∗ relaxation times in the same areas as visually observed hypointense areas and for the same amount of time after injection. This is depicted in Figure 6, which consists of T2∗ relaxation time difference bullseye plots from the same patient as used in Figure 4. In Figure 6 plots, the areas of decreased T2∗ relaxation times are seen in the same areas as the hypointense areas in Figure 4.

In Figure 6, we also see the NOGA injection map from the same patient. The four yellow points in the map show the injection points for the USPIO-labeled cells. The location of these points correlates with the hypointense areas seen in Figure 4 and with the decreased T2∗ relaxation times in Figure 6.

## 4. Discussion

This was a first-in-man clinical experience aimed at evaluating the utility of MRI tracking of USPIO-labeled bone marrow-derived autologous MSCs after intramyocardial injection in patients with chronic IHD.

The primary objective of the study was *in vivo* visualization of USPIO-labeled MSCs using MRI at day 0 after injection of cells. This was achieved successfully. Secondary objectives included visualization of labeled cells at later time points. We were able to visualize the labeled cells for 1 to 14 days after injection of cells. During this period, the signal from the labeled cells gradually decreased. Hereafter, we could not distinguish labeled cells from background noise on the MRI images.

We can only speculate if this is due to migration and dispersion of the cells to the rest of the myocardium or if most of the injected cells are washed out into the peripheral circulation. A combination of both mechanisms is perhaps the most likely scenario. Another possibility is that the cells died and the label was phagocytosed. This phenomenon was demonstrated in two preclinical studies performed in rats after acute MI. In both studies, iron oxide-labeled cells were tracked for 3-4 weeks, but postmortem histology analyses showed that the cells were engulfed in macrophages that had infiltrated the injection areas [21, 22]. In contrast, 10 other preclinical studies demonstrated the opposite. These animal studies were carried out in rats, mice, canines, and pigs after acute MI, and cells were identified using both MRI and postmortem histologic analysis [11–20]. In these studies, iron oxide-labeled cells were identified using these methods for longer periods, ranging from 3 to 16 weeks after transplantation. Macrophage-specific CD68 staining was done and showed no macrophages were present in the cardiac tissue containing USPIO cells, and moreover, the USPIO particles were contained in the original labeled cells. One explanation for these differences could be that the cells were injected into the infarct area in the first-mentioned studies in contrast to the latter 10 mentioned studies, where cells were injected into the border zone of the infarction. Therefore, we do not believe that the cells and USPIO particles in the present study were engulfed in macrophages.

One of the differences between the animal studies and the present study is that the present study was carried out in patients with chronic IHD. Another significant difference between the animal studies and the present clinical study is the potential to carry out postmortem histologic analyses in the animal studies, which substantially strengthened these studies. Furthermore, coregistration of reducing hypointense areas temporally on MRI images with end-point histological analysis confirms that these hypointense areas on these images represent labeled cells.

In addition to visual evaluation of MRI images, we have determined changes in T2∗ relaxation times regionally, but after a few days, the background noise in the T2∗ images was indistinguishable from potential signal from labeled cells. The reduction of the MRI signal over time may be due to both migration and dispersion of the MSCs from the site of injection within the myocardium and/or washout of MSCs from the heart by the myocardial perfusion.

The mechanisms behind the regenerative capacity of MSCs are not fully understood. However, most recent evidence suggests that secretion of cytokines and growth factors from the MSCs is as the main mechanism [41–44].

In the future, for studies to improve detectability, we suggest using double labeling with both iron oxide particles and a PET-tracer and perform the tracking using combined PET-MRI scanners, which is now possible. The MRI scanners used in the animal studies varied in field strength from 1.5 to 11.7 T, and another option for future studies could be the use of MRI scanners with higher field strength, although the higher signal to noise ratio is accompanied with a higher degree of image artifacts when using MRI scanners with higher field strengths [45, 46].

The labeling of MSCs with iron oxide particles does not seem to have any adverse effects on myocardial function.

In conclusion, we have demonstrated that in vivo MRI tracking of intramyocardial injected MSCs labeled with USPIO particles is feasible and safe. We were able to detect MRI signal from the USPIO particles up to 2 weeks after transplantation. In clinical trials, postmortem histological analysis of the heart is not an option, and therefore, we cannot guarantee whether the detected USPIO particles in this study were in fact still within the original labeled MSCs. However, most preclinical animal studies have demonstrated that USPIOs do in fact remain within the MSC and are not engulfed by macrophages. Therefore, we do believe that the demonstrated MRI signal in this study originated from USPIO-labeled MSCs. After 1 month, it was no longer possible to distinguish labeled cells from background noise. Whether this was due to migration and dispersion of the cells to the rest of the myocardium or if injected cells are washed out into the peripheral circulation remains to be clarified. However, improved heart function was observed in all patients posttransplantation. Dual labeling with both USPIO particles and radioisotopes and tracking with combined PET-MRI scanners could help clarify this in the future, especially when radioisotopes with longer half-times than presently available are approved. Better T2∗ protocols might also reduce the amount of background noise and thus allow for tracking lower concentrations of labeled cells.

## Data Availability

The data used to support the findings of this study are restricted by the Danish National Ethical Committee in order to protect patient privacy. Data are available from the corresponding author for researchers who meet the criteria for access to confidential data.

## Abbreviations

CABG: Coronary artery bypass grafting
CCS: Canadian Cardiovascular Society
IHD: Ischemic heart disease
LV: Left ventricle
LVEDV: Left ventricle end-diastolic volume
LVEF: Left ventricle ejection fraction
LVESV: Left ventricle end-systolic volume
MI: Myocardial infarction
MRI: Magnetic resonance imaging
MSC: Mesenchymal stromal cell
NYHA: New York Heart Association
PET: Positron emission tomography
PCI: Percutaneous coronary intervention
SAQ: Seattle Angina Questionnaire
SV: Stroke volume
USPIO: Ultrasmall paramagnetic iron oxide.

## Supplementary Materials

Supplementary Materials Table S1: inclusion and exclusion criteria. Detailed inclusion and exclusion criteria for the study. NYHA = New York Heart Association; CCS = Canadian Cardiovascular Society; PCI = percutaneous coronary intervention; CABG = coronary artery bypass grafting; COPD = chronic obstructive pulmonary disease; FEV1 = forced expiratory volume during first second; CKMB = creatine kinase myocardial band; TCI = transitory cerebral ischemia; ICD = implantable cardioverter defibrillator. Table S2: summary of events. Overview of all study-related procedures and events during the study period. NYHA = New York Heart Association; CCS = Canadian Cardiovascular Society; SAQ = Seattle Angina Questionnaire; MRI = magnetic resonance imaging; MSC = mesenchymal stromal cell; USPIO = ultrasmall paramagnetic iron oxide. Table S3: baseline characteristics. Detailed baseline characteristics of the patients included in the study. SD = standard deviation; BMI = body mass index; LVEF = left ventricular ejection fraction; NYHA = New York Heart Association; CCS = Canadian Cardiovascular Society; MI = myocardial Infarction; PCI = percutaneous coronary intervention; CABG = coronary artery bypass grafting. Values are mean ± SD or number (%). Table S4: supplemental results. This table contains clinical results in regard to cardiac pump function and patient clinical symptoms and quality of life. EDV = end-diastolic volume; ESV = end-systolic volume; LVEF = left ventricular ejection fraction; SV = stroke volume; NYHA = New York Heart Association; CCS = Canadian Cardiovascular Society; SAQ = Seattle Angina Questionnaire; QoL = quality of life. All values are mean ± standard deviation.

## References

[1] Fisher S. A., Zhang H., Doree C., Mathur A., Martin-Rendon E. (2015). Stem cell treatment for acute myocardial infarction. *Cochrane Database of Systematic Reviews*.

[2] Fisher S. A., Doree C., Mathur A., Taggart D. P., Martin-Rendon E. (2016). Stem cell therapy for chronic ischaemic heart disease and congestive heart failure. *Cochrane Database of Systematic Reviews*.

[3] Heldman A. W., DiFede D. L., Fishman J. E. (2014). Transendocardial mesenchymal stem cells and mononuclear bone marrow cells for ischemic cardiomyopathy: the TAC-HFT randomized trial. *JAMA*.

[4] Hare J. M., Fishman J. E., Gerstenblith G. (2012). Comparison of allogeneic vs autologous bone marrow–derived mesenchymal stem cells delivered by transendocardial injection in patients with ischemic cardiomyopathy: the POSEIDON randomized trial. *JAMA*.

[5] Perin E. C., Borow K. M., Silva G. V. (2015). A phase II dose-escalation study of allogeneic mesenchymal precursor cells in patients with ischemic or nonischemic heart failure. *Circulation Research*.

[6] Kastrup J., Haack-Sørensen M., Juhl M. (2017). Cryopreserved off‐the‐shelf allogeneic adipose‐derived stromal cells for therapy in patients with ischemic heart disease and heart failure—a safety study. *Stem Cells Translational Medicine*.

[7] Mathiasen A. B., Qayyum A. A., Jørgensen E. (2015). Bone marrow-derived mesenchymal stromal cell treatment in patients with severe ischaemic heart failure: a randomized placebo-controlled trial (MSC-HF trial). *European Heart Journal*.

[8] Hare J. M., DiFede D. L., Rieger A. C. (2017). Randomized comparison of allogeneic versus autologous mesenchymal stem cells for nonischemic dilated cardiomyopathy: POSEIDON-DCM trial. *Journal of the American College of Cardiology*.

[9] Mathiasen A. B., Kastrup J. (2013). Non-invasive in-vivo imaging of stem cells after transplantation in cardiovascular tissue. *Theranostics*.

[10] Zhang W. Y., Ebert A. D., Narula J., Wu J. C. (2011). Imaging cardiac stem cell therapy: translations to human clinical studies. *Journal of Cardiovascular Translational Research*.

[11] Stuckey D. J., Carr C. A., Martin-Rendon E. (2006). Iron particles for noninvasive monitoring of bone marrow stromal cell engraftment into, and isolation of viable engrafted donor cells from, the heart. *Stem Cells*.

[12] Ebert S. N., Taylor D. G., Nguyen H. L. (2007). Noninvasive tracking of cardiac embryonic stem cells in vivo using magnetic resonance imaging techniques. *Stem Cells*.

[13] Chapon C., Jackson J. S., Aboagye E. O., Herlihy A. H., Jones W. A., Bhakoo K. K. (2009). An in vivo multimodal imaging study using MRI and PET of stem cell transplantation after myocardial infarction in rats. *Molecular Imaging and Biology*.

[14] Bulte J. W., Kostura L., Mackay A. (2005). Feridex-labeled mesenchymal stem cells: cellular differentiation and MR assessment in a canine myocardial infarction model. *Academic Radiology*.

[15] Kraitchman D. L., Heldman A. W., Atalar E. (2003). In vivo magnetic resonance imaging of mesenchymal stem cells in myocardial infarction. *Circulation*.

[16] Amado L. C., Saliaris A. P., Schuleri K. H. (2005). Cardiac repair with intramyocardial injection of allogeneic mesenchymal stem cells after myocardial infarction. *Proceedings of the National Academy of Sciences of the United States of America*.

[17] Ma G. S., Qi C. M., Liu N. F. (2011). Efficiently tracking of stem cells in vivo using different kinds of superparamagnetic iron oxide in swine with myocardial infarction. *Chinese Medical Journal*.

[18] Peng C., Yang K., Xiang P. (2013). Effect of transplantation with autologous bone marrow stem cells on acute myocardial infarction. *International Journal of Cardiology*.

[19] Yang K., Xiang P., Zhang C. (2011). Magnetic resonance evaluation of transplanted mesenchymal stem cells after myocardial infarction in swine. *Canadian Journal of Cardiology*.

[20] Graham J. J., Foltz W. D., Vaags A. K. (2010). Long-term tracking of bone marrow progenitor cells following intracoronary injection post-myocardial infarction in swine using MRI. *American Journal of Physiology-Heart and Circulatory Physiology*.

[21] Amsalem Y., Mardor Y., Feinberg M. S. (2007). Iron-oxide labeling and outcome of transplanted mesenchymal stem cells in the infarcted myocardium. *Circulation*.

[22] Terrovitis J., Stuber M., Youssef A. (2008). Magnetic resonance imaging overestimates ferumoxide-labeled stem cell survival after transplantation in the heart. *Circulation*.

[23] Richards J. M. J., Shaw C. A., Lang N. N. (2012). In vivo mononuclear cell tracking using superparamagnetic particles of iron oxide: feasibility and safety in humans. *Circulation: Cardiovascular Imaging*.

[24] Callera F., de Melo C. M. T. P. (2007). Magnetic resonance tracking of magnetically labeled autologous bone marrow CD34^+^ cells transplanted into the spinal cord via lumbar puncture technique in patients with chronic spinal cord injury: CD34^+^ cells’ migration into the injured site. *Stem Cells and Development*.

[25] de Vries I. J. M., Lesterhuis W. J., Barentsz J. O. (2005). Magnetic resonance tracking of dendritic cells in melanoma patients for monitoring of cellular therapy. *Nature Biotechnology*.

[26] Karussis D., Karageorgiou C., Vaknin-Dembinsky A. (2010). Safety and immunological effects of mesenchymal stem cell transplantation in patients with multiple sclerosis and amyotrophic lateral sclerosis. *Archives of Neurology*.

[27] Toso C., Vallee J.-P., Morel P. (2008). Clinical magnetic resonance imaging of pancreatic islet grafts after iron nanoparticle labeling. *American Journal of Transplantation*.

[28] Zhu J., Zhou L., XingWu F. (2006). Tracking neural stem cells in patients with brain trauma. *New England Journal of Medicine*.

[29] Nighoghossian N., Wiart M., Cakmak S. (2007). Inflammatory response after ischemic stroke: a USPIO-enhanced MRI study in patients. *Stroke*.

[30] Bulte J. W. M. (2009). In vivo MRI cell tracking: clinical studies. *American Journal of Roentgenology*.

[31] Song M., Moon W. K., Kim Y., Lim D., Song I. C., Yoon B. W. (2007). Labeling efficacy of superparamagnetic iron oxide nanoparticles to human neural stem cells: comparison of ferumoxides, monocrystalline iron oxide, cross-linked iron oxide (CLIO)-NH2 and tat-CLIO. *Korean Journal of Radiology*.

[32] Bonnemain B. (2008). Nanoparticles: the industrial viewpoint. Applications in diagnostic imaging. *Annales Pharmaceutiques Françaises*.

[33] Wunderbaldinger P., Josephson L., Weissleder R. (2002). Crosslinked iron oxides (CLIO): a new platform for the development of targeted MR contrast agents. *Academic Radiology*.

[34] Josephson L., Tung C. H., Moore A., Weissleder R. (1999). High-efficiency intracellular magnetic labeling with novel superparamagnetic-tat peptide conjugates. *Bioconjugate Chemistry*.

[35] Hansen L., Hansen A. B., Mathiasen A. B. (2014). Ultrastructural characterization of mesenchymal stromal cells labeled with ultrasmall superparamagnetic iron-oxide nanoparticles for clinical tracking studies. *Scandinavian Journal of Clinical and Laboratory Investigation*.

[36] Mathiasen A. B., Hansen L., Friis T., Thomsen C., Bhakoo K., Kastrup J. (2013). Optimal labeling dose, labeling time, and magnetic resonance imaging detection limits of ultrasmall superparamagnetic iron-oxide nanoparticle labeled mesenchymal stromal cells. *Stem Cells International*.

[37] Haack-Sørensen M., Hansen S. K., Hansen L. (2013). Mesenchymal stromal cell phenotype is not influenced by confluence during culture expansion. *Stem Cell Reviews and Reports*.

[38] Friis T., Haack-Sørensen M., Mathiasen A. B. (2011). Mesenchymal stromal cell derived endothelial progenitor treatment in patients with refractory angina. *Scandinavian Cardiovascular Journal*.

[39] Dominici M., Le Blanc K., Mueller I. (2006). Minimal criteria for defining multipotent mesenchymal stromal cells. The International Society for Cellular Therapy position statement. *Cytotherapy*.

[40] Kastrup J., Jorgensen E., Ruck A. (2005). Direct intramyocardial plasmid vascular endothelial growth factor- A_165_gene therapy in patients with stable severe angina pectoris: A randomized double-blind placebo-controlled study: The Euroinject One trial. *Journal of the American College of Cardiology*.

[41] Gnecchi M., Zhang Z., Ni A., Dzau V. J. (2008). Paracrine mechanisms in adult stem cell signaling and therapy. *Circulation Research*.

[42] Hatzistergos K. E., Quevedo H., Oskouei B. N. (2010). Bone marrow mesenchymal stem cells stimulate cardiac stem cell proliferation and differentiation. *Circulation Research*.

[43] Sassoli C., Pini A., Mazzanti B. (2011). Mesenchymal stromal cells affect cardiomyocyte growth through juxtacrine Notch-1/Jagged-1 signaling and paracrine mechanisms: clues for cardiac regeneration. *Journal of Molecular and Cellular Cardiology*.

[44] Timmers L., Lim S. K., Hoefer I. E. (2011). Human mesenchymal stem cell-conditioned medium improves cardiac function following myocardial infarction. *Stem Cell Research*.

[45] Nguyen K.-L., Khan S. N., Moriarty J. M. (2015). High-field MR imaging in pediatric congenital heart disease: initial results. *Pediatric Radiology*.

[46] Meloni A., Hezel F., Positano V. (2014). Detailing magnetic field strength dependence and segmental artifact distribution of myocardial effective transverse relaxation rate at 1.5, 3.0, and 7.0 T. *Magnetic Resonance in Medicine*.

