# Supplementary material for: In Vivo MRI Tracking of Mesenchymal Stromal Cells Labeled with Ultrasmall Paramagnetic Iron Oxide Particles after Intramyocardial Transplantation in Patients with Chronic Ischemic Heart Disease
Source: Stem Cells Int. 2019 Nov 14;2019:2754927. doi: 10.1155/2019/2754927 (PMC6877937; doi:10.1155/2019/2754927)
Supplement: Supplementary Materials — Table S1: inclusion and exclusion criteria. Detailed inclusion and exclusion criteria for the study. NYHA = New York Heart Association; CCS = Canadian Cardiovascular Society; PCI = percutaneous coronary intervention; CABG = coronary artery bypass grafting; COPD = chronic obstructive pulmonary disease; FEV1 = forced expiratory volume during first second; CKMB = creatine kinase myocardial band; TCI = transitory cerebral ischemia; ICD = implantable cardioverter defibrillator. Table S2: summary of events. Overview of all study-related procedures and events during the study period. NYHA = New York Heart Association; CCS = Canadian Cardiovascular Society; SAQ = Seattle Angina Questionnaire; MRI = magnetic resonance imaging; MSC = mesenchymal stromal cell; USPIO = ultrasmall paramagnetic iron oxide. Table S3: baseline characteristics. Detailed baseline characteristics of the patients included in the study. SD = standard deviation; BMI = body mass index; LVEF = left ventricular ejection fraction; NYHA = New York Heart Association; CCS = Canadian Cardiovascular Society; MI = myocardial Infarction; PCI = percutaneous coronary intervention; CABG = coronary artery bypass grafting. Values are mean ± SD or number (%). Table S4: supplemental results. This table contains clinical results in regard to cardiac pump function and patient clinical symptoms and quality of life. EDV = end-diastolic volume; ESV = end-systolic volume; LVEF = left ventricular ejection fraction; SV = stroke volume; NYHA = New York Heart Association; CCS = Canadian Cardiovascular Society; SAQ = Seattle Angina Questionnaire; QoL = quality of life. All values are mean ± standard deviation. [file 2754927.f1.pdf]

Supplemental Material

Table S1: Inclusion and exclusion criteria

Inclusion criteria

- Age between 30 and 80 years.
- Signed informed consent.
- Chronic stable ischemic heart disease
- NYHA class II-IV or CCS class II-IV
- Maximal tolerable angina and/or heart failure medication.
- Angiography within 12 months of inclusion. Angiography must have at least one larger coronary vessel with a significant stenosis with no option for PCI or CABG (Angiographies evaluated by an independent thoracic surgeon and an interventional cardiologist).
- Patients who have had PCI or CABG within 6 months of inclusion must have a new angiography at least 4 months after the intervention to rule out early restenosis.

Exclusion criteria

- Pregnant or fertile women.
- Clinical significant anemia, leukopenia, leukocytosis or thrombocythemia.
- Diminished functional capacity for other reasons such as: COPD with FEV1<1 L/min, moderate to severe claudication or morbid obesity.
- Patients with reduced immune response or treated with immunosuppressive medication.
- Moderate to severe valvular disease or valvular disease with option for valvular surgery.
- Acute coronary syndrome with elevation of CKMB or troponins, stroke or TCI within 6 weeks of inclusion.
- History with malignant disease within 5 years of inclusion or suspected malignity.
- Other experimental treatment within 4 weeks of baseline evaluation.
- Other revascularization treatment within 4 months of treatment.
- Contraindications for MRI such as: Claustrophobia, pacemaker, ICD unit, metal fragments or metal implants in the cranium.

NYHA = New York Heart Association, CCS = Canadian Cardiovascular Society, PCI = percutaneous coronary intervention, CABG = coronary artery bypass grafting, COPD = chronic obstructive pulmonary disease, FEV1 = forced expiratory volume during first second, CKMB = creatine kinase myocardial band, TCI = Transitory cerebral ischemia, ICD = implantable cardioverter defibrillator.

Table S2: Summary of events

	Baseline	D-1	D0	D1	D3	D7	W2	W4	W8	W12	W26
Informed consent	●										
History / physical exam	●	●		●	●	●	●	●	●	●	●
Laboratories	●	●	●	●				●	●	●	●
NYHA	●							●	●	●	●
CCS	●							●	●	●	●
SAQ	●									●	●
MRI	●		●	●	●	●	●	●	●	●	●
Bone marrow aspiration	●										
MSC isolation and expansion	●										
USPIO labeling		●									
NOGA Mapping			●								
Holter monitoring		●	●	●							
Adverse events evaluation			●	●	●	●	●	●	●	●	●

NYHA = New York Heart Association, CCS = Canadian Cardiovascular Society, SAQ = Seatlle Angina Questionnaire, MRI = Magnetic resonance imaging, MSC = Mesenchymal stromal cell, USPIO = Ultra-small paramagnetic iron-oxide

Table S3: Baseline Characteristics

Parameter	Data
Age (years)	62 ± 7
Sex (male)	5 (100)
BMI (kg/m2)	28.7 ± 3.4
LVEF (%)	41.6 ± 6.6
NYHA Class	2.2 ± 0.4
CCS Class	1.8 ± 0.4
Previous MI	5 (100)
Previous PCI	5 (100)
Previous CABG	5 (100)
Diabetes	3 (60)
Hypertension	4 (80)

SD = standard deviation, BMI = body mass index, LVEF = left ventricular ejection fraction, NYHA = New York Heart Association, CCS = Canadian Cardiovascular Society, MI = myocardial Infarction, PCI = percutaneous coronary intervention, CABG = coronary artery by-pass grafting

Values are mean ± SD or number (%).

Table S4: Supplemental results

Parameter	Baseline	3 months	6 months	p-value
EDV (mL)	189 ± 29	184 ± 20	183 ± 21	0.55
ESV (mL)	111 ± 21	102 ± 19	90 ± 22	0.007
LVEF (%)	41.5 ± 6.7	44.6 ± 8.2	50.8 ± 9.4	0.015
SV (mL)	78 ± 17	82 ± 17	93 ± 18	0.07
CCS Class	1.8 ± 0.4	0.4 ± 0.5	0.2 ± 0.4	0.009
NYHA Class	2.2 ± 0.4	1.8 ± 0.8	1.6 ± 0.9	0.10
SAQ – QoL score	57 ± 19	65 ± 27	70 ± 22	0.14

EDV = end diastolic volume, ESV = end systolic volume, LVEF = left ventricular ejection fraction, SV = stroke volume, NYHA = New York Heart Association, CCS = Canadian Cardiovascular Society, SAQ = Seattle Angina Questionnaire, QoL = quality of life

All values are mean ± standard deviation.
